# An evaluation of mathematics teachers’ beliefs, teaching methods, and competencies regarding students with mathematics learning difficulties

**DOI:** 10.3389/fpsyg.2026.1945419

**Published:** 2026-10-14

**Authors:** Cahit Taşdemir

**Affiliations:** Tatvan Vocational School, Bitlis Eren University, Bitlis, Türkiye

**Keywords:** instructional strategies, mathematics achievement, mathematics learning difficulties, mathematics teachers, students with learning disabilities, teacher self-efficacy

## Abstract

Students who struggle with mathematics generally face ongoing difficulties in understanding mathematical terms, applying operational knowledge, and developing problem-solving skills. In this process, teachers’ attitudes, teaching methods, and professional competence play a significant role in shaping students’ mathematics learning experiences. This study aims to examine mathematics teachers’ perceptions, teaching methods, and the challenges they face regarding students with mathematics learning difficulties, based on their professional experiences. The research was conducted using a qualitative case study design. The participants consisted of 20 middle school mathematics teachers working in public middle schools in Türkiye’s Eastern Anatolia Region. The data were obtained through semi-structured interviews and evaluated using the content analysis technique. The findings revealed that teachers identified difficulties in mathematics learning based on the retention of prior knowledge, problem-solving skills, conceptual understanding, and deficiencies in basic mathematical operations. The teachers also noted that negative attitudes toward mathematics, lack of motivation, and low self-confidence had negative repercussions on students’ learning processes. In terms of teaching methods, the teachers generally employed approaches such as concrete materials, personalized instruction, technology-supported activities, and peer-assisted learning to strengthen students’ mathematics skills. Nevertheless, many teachers point out that they lack the necessary knowledge, professional training, and self-confidence to provide effective and systematic support to students who struggle with mathematics. Research findings highlight the importance of assessing mathematics learning difficulties from a multifaceted perspective that considers a combination of cognitive, emotional, and instructional factors. Furthermore, strengthening practice-oriented professional development programs can enhance teachers’ competencies and contribute to a more inclusive and effective mathematics education.

## 1 Introduction

Mathematics learning constitutes a fundamental component of students’ academic development because it involves the coordinated use of number knowledge, calculation, conceptual understanding, reasoning, and problem solving. However, students do not acquire mathematical knowledge and skills at the same pace. Some students experience persistent difficulties in acquiring, understanding, retaining, and applying mathematical knowledge, while others show lower achievement that may be associated with instructional, motivational, linguistic, environmental, or other contextual factors. Contemporary research emphasizes that mathematics learning difficulties are heterogeneous rather than representing a single, uniform learning profile (Geary, 2013; Nelson et al., 2022). Difficulties in mathematics may involve not only numerical and conceptual processing but also affective and motivational factors, including mathematics anxiety, reduced confidence, and negative attitudes toward mathematics (Geary, 2013; Gunderson et al., 2017; Ramirez et al., 2018).

The literature has also demonstrated that mathematics difficulties may involve different domains of mathematical knowledge. Difficulties may become evident in basic number knowledge and arithmetic operations, calculation fluency, mathematical reasoning, fractions, word-problem solving, and the understanding and application of mathematical concepts. A systematic review of research syntheses found that intervention research on mathematics learning difficulties has particularly emphasized word-problem solving, fractions, computer-assisted learning, and schema-based instruction, while also reporting substantial variation in how mathematics learning disabilities and mathematics difficulties are defined (Nelson et al., 2022). Similarly, research focusing specifically on middle-school students has demonstrated that students experiencing mathematics difficulty can benefit from a range of targeted instructional interventions (Powell et al., 2021).

Importantly, mathematics learning difficulties should not be equated automatically with low mathematics achievement or with a clinically diagnosed mathematics learning disability. Although these groups may overlap in observed mathematical performance, research has identified differences between students with mathematical learning disabilities and students who are low achieving in mathematics. For example, longitudinal research has shown that mathematical learning disability and persistent low achievement can constitute distinguishable developmental profiles, although they may share some cognitive risk factors (Zhang et al., 2018). A recent systematic review likewise reported that students with mathematical learning disabilities tend to show broader and more pronounced cognitive weaknesses than low-achieving students, although both groups may experience difficulties in mathematical performance (Kivirähk-Koor and Kiive, 2025). Therefore, educational research needs to distinguish between clinically or psychometrically defined disability categories and the broader classroom phenomenon of students experiencing substantial difficulties in mathematics.

A further complexity concerns the developmental and multidimensional nature of mathematics learning difficulties. Contemporary accounts increasingly reject the assumption that a single cognitive deficit can explain all forms of mathematical difficulty. A multi-deficit perspective proposes that mathematical difficulties may emerge from the interaction of domain-specific and domain-general cognitive processes. Difficulties in number processing may coexist with weaknesses in working memory, processing speed, language, attention, or other cognitive processes. Such factors may also contribute to the co-occurrence of mathematics difficulties with difficulties in reading or other learning domains. Consequently, students experiencing mathematics difficulties may demonstrate different patterns of strengths and weaknesses rather than a single characteristic profile.

The classroom context is therefore particularly important. Mathematics teachers are required to interpret students’ mathematical performance, identify areas of difficulty, and select instructional responses within the constraints of ordinary classroom settings. Teachers’ beliefs and perceptions can constitute an important interpretive dimension of this process because they influence how teachers conceptualize students’ learning needs and their own instructional responsibilities. Research on inclusive mathematics education has increasingly emphasized the importance of teachers’ beliefs, attitudes, and self-efficacy for developing inclusive teaching practices (Nührenbörger et al., 2025; Scherer and Bertram, 2024). Recent research has also shown that professional learning experiences can influence teachers’ attitudes and self-efficacy expectations concerning inclusive mathematics instruction (Nührenbörger et al., 2025).

Despite this growing literature, an important gap remains. Existing research has extensively examined the cognitive characteristics of mathematics learning difficulties and the effectiveness of particular interventions, but comparatively less qualitative attention has been devoted to how practicing middle-school mathematics teachers interpret students’ persistent mathematics difficulties and how those interpretations are reflected in their reported classroom practices. This issue is particularly important because teachers encounter students with diverse mathematical profiles within regular classrooms and must make instructional decisions without necessarily having access to formal diagnostic information.

Accordingly, the present study focuses on mathematics teachers rather than students as the primary source of data. Specifically, the study examines how middle-school mathematics teachers perceive students whom they identify as experiencing substantial and persistent difficulties in mathematics, how they describe these students’ mathematical and affective challenges, which instructional approaches they report using, and how they perceive their own competence and professional preparation for supporting these students.

## 2 Literature review

### 2.1 Conceptualizing mathematics learning difficulties

Mathematics learning difficulties (MLD) represent a heterogeneous area of research in which terminology and identification criteria vary across disciplines. Terms such as mathematics difficulty, mathematics learning disability, mathematical learning disorder, persistent low achievement, and developmental dyscalculia have not always been used consistently. A state-of-the-art review of research in mathematics education demonstrated considerable variation in how MLD has been conceptualized and described across the literature (Deruaz et al., 2020). More recent systematic evidence similarly indicates substantial variation in operational definitions of mathematics learning disability and mathematics difficulty across intervention research (Nelson et al., 2022).

One influential theoretical perspective conceptualizes MLD from a multi-deficit framework. Rather than attributing mathematical difficulties to a single underlying deficit, this perspective assumes that mathematical learning may be affected by the interaction of multiple domain-specific and domain-general processes. Domain-specific processes may involve numerical magnitude processing, number representation, calculation, and arithmetic knowledge, whereas domain-general processes may include working memory, processing speed, attention, language, and reasoning. From this perspective, students with MLD may display different combinations of cognitive and mathematical strengths and weaknesses rather than a homogeneous profile.

This theoretical perspective is particularly relevant to the present study because teachers may observe different manifestations of mathematical difficulty in classroom contexts. For example, one student may experience substantial difficulty with basic operations and calculation fluency, whereas another may demonstrate relatively adequate procedural knowledge but struggle with conceptual understanding, mathematical reasoning, or problem solving. Consequently, classroom perceptions of mathematics learning difficulties should not be reduced to a single indicator such as examination performance.

Mathematical difficulties may also become evident across different mathematical content domains. Research has documented difficulties in number knowledge, calculation, arithmetic fluency, fractions, algebraic concepts, and mathematical word problems. Nelson et al. (2022) reported that research syntheses have particularly emphasized word-problem solving, fractions, and computer-assisted mathematics interventions. Research focusing on middle-school students has similarly examined interventions addressing a broad range of mathematical content and instructional processes (Powell et al., 2021).

Another important characteristic is developmental change. Mathematics learning is cumulative, meaning that weaknesses in foundational number knowledge and calculation may affect the acquisition of increasingly complex mathematical concepts. Longitudinal evidence indicates that persistent mathematical difficulties can be identified across several years of schooling, while different developmental profiles may emerge among students with mathematics learning disability and persistent low achievement (Zhang et al., 2018). Therefore, MLD should be understood as a developmental phenomenon that may change in its manifestation as students encounter increasingly complex mathematical demands.

MLD may also co-occur with difficulties in other learning domains. The multiple-deficit perspective suggests that some cognitive risk factors are domain-specific, whereas others may affect several learning domains. For example, working memory, processing speed, and language-related processes may contribute to difficulties across mathematics and reading. Recent evidence has continued to demonstrate the relevance of co-occurring mathematics and reading difficulties, indicating that students with difficulties in both domains may experience more extensive cognitive and academic challenges.

#### 2.1.1 Distinguishing mathematics learning difficulties from low achievement

An important conceptual distinction in the present study is the difference between mathematics learning difficulties and general low mathematics achievement. Low achievement may result from numerous factors, including limited instructional opportunities, absenteeism, socioeconomic conditions, motivational factors, language-related difficulties, insufficient prior learning, or other contextual influences. In contrast, mathematics learning disability generally refers to more persistent and specific difficulties that cannot be explained solely by general educational disadvantage.

Longitudinal research has demonstrated that mathematics learning disability and persistent low achievement can be distinguished empirically, although they may share some underlying cognitive characteristics (Zhang et al., 2018). Recent systematic evidence likewise suggests that students with mathematical learning disabilities generally exhibit more pronounced cognitive weaknesses than low-achieving students (Kivirähk-Koor and Kiive, 2025).

The distinction is particularly important for the present study because the participants were mathematics teachers rather than diagnosticians, and the study did not include standardized diagnostic assessments of students. Accordingly, the term “mathematics learning difficulties” is used in this study to refer to teachers’ perceptions of students who demonstrate substantial and persistent difficulties in learning mathematics in their classroom experience. It does not imply that the participating teachers clinically diagnosed these students with dyscalculia or a formally recognized specific learning disability.

#### 2.1.2 Operational definition used in the present study

For the purposes of this study, mathematics learning difficulties are defined as persistent and observable difficulties in acquiring, understanding, retaining, and applying mathematical knowledge and skills, as perceived and described by practicing middle-school mathematics teachers. The term does not imply a clinical diagnosis of dyscalculia or a formally diagnosed specific learning disorder. Rather, it refers to students whom participating teachers identify as experiencing substantial and persistent difficulties in mathematics within inclusive classroom settings.

This operational definition is deliberately broader than a clinical definition of mathematical learning disability because the present study investigates teachers’ classroom perceptions rather than diagnostic status.

### 2.2 Variation in the identification and prevalence of mathematics learning difficulties

Reported prevalence rates of mathematics learning difficulties vary considerably across studies. Such variation should not necessarily be interpreted as evidence that the actual prevalence of MLD differs substantially across populations. Instead, prevalence estimates are influenced by how mathematics difficulty is defined, which mathematical domains are assessed, the age or grade level of participants, the assessment instruments used, the statistical thresholds applied, and whether the study examines mathematics difficulty, low achievement, or clinically defined mathematics learning disability. Nelson et al. (2022) demonstrated this definitional heterogeneity in their systematic review of research syntheses. Their review included 36 research syntheses comprising 836 studies and 32,495 participants and found substantial variation in the operational definitions used to identify learning disabilities, mathematics learning disabilities, and mathematics difficulties.

This variability has important implications for educational research. A student who performs below expectations in mathematics is not necessarily experiencing a specific learning disability. Conversely, students with persistent and specific mathematics difficulties may not always be formally identified through clinical or psychometric procedures. Therefore, classroom-based research should clearly specify whether the term refers to diagnosed disability, persistent low achievement, mathematics difficulty, or teachers’ perceptions of students experiencing difficulty. The present study adopts the latter perspective. It does not attempt to estimate the prevalence of MLD among students. Instead, it examines how teachers recognize and interpret mathematics learning difficulties within their everyday classroom experiences.

### 2.3 Mathematics teachers’ beliefs and perceptions of students experiencing mathematics learning difficulties

Teachers’ beliefs and perceptions have long been recognized as important components of teachers’ professional cognition. Rather than representing isolated opinions, teacher beliefs may include assumptions about students’ abilities, the nature of mathematics learning, effective instruction, inclusion, and teachers’ own professional capabilities (Pajares, 1992).

Within inclusive mathematics education, teachers’ beliefs are particularly relevant because teachers must respond to heterogeneous learners while maintaining access to common curricular goals. Recent research indicates that teachers’ attitudes and self-efficacy expectations are relevant to inclusive mathematics instruction and can be influenced by professional learning experiences (Nührenbörger et al., 2025). Similarly, research on professionalization for inclusive mathematics education has emphasized the relationship between teacher education, beliefs, and self-efficacy (Scherer and Bertram, 2024). According to Bandura (1997, p.3) perceived self-efficacy refers to “beliefs in one’s capabilities to organize and execute the courses of action required to produce given attainments.

Teachers’ perceptions of students with mathematics learning difficulties may operate at several interconnected levels. First, teachers interpret students’ observable mathematical performance, such as difficulties with basic operations, conceptual understanding, mathematical reasoning, or problem solving. Second, they may interpret affective and motivational characteristics, such as confidence, motivation, persistence, or attitudes toward mathematics. Third, teachers make judgments about what kinds of support students require and what instructional strategies may be feasible within the classroom.

These perceptions should not, however, be understood as direct determinants of student achievement. Rather, they constitute one component of teachers’ professional decision-making. The significance of studying teacher perceptions lies in understanding how teachers make sense of students’ difficulties and how these understandings are connected to the instructional responses they report.

This connection is particularly important because research on mathematics interventions demonstrates that students experiencing mathematics difficulties can benefit from targeted instructional approaches. Systematic evidence from middle-school mathematics research indicates positive effects for a substantial proportion of investigated interventions, although effects vary according to content, instructional approach, and learner characteristics (Powell et al., 2021). Consequently, teachers need not only to recognize mathematical difficulties but also to possess sufficient pedagogical knowledge and confidence to select appropriate responses.

Teacher self-efficacy is relevant within this context. Self-efficacy refers to teachers’ beliefs concerning their capabilities to organize and execute actions required to accomplish particular teaching tasks. In inclusive mathematics education, teachers’ perceived efficacy may influence how prepared they feel to respond to diverse learner needs. Recent intervention research has shown that structured professional learning can support teachers’ attitudes and self-efficacy expectations concerning inclusive mathematics teaching (Nührenbörger et al., 2025).

Accordingly, the present study conceptualizes teacher perceptions broadly, encompassing teachers’ interpretations of students’ mathematical and affective difficulties, their beliefs concerning instructional needs, their reported instructional responses, and their perceived competence and preparedness. This conceptualization allows the study to examine not simply whether teachers perceive students as having difficulties, but also how those perceptions are translated into reported classroom practices.

### 2.4 Theoretical perspectives on pedagogical responses to mathematics learning difficulties

Supporting students who experience mathematics learning difficulties requires instructional responses that are sensitive to both the mathematical content and the learner’s current level of understanding. The intervention literature indicates that no single instructional technique is sufficient for all students because mathematical difficulties are heterogeneous.

One important principle is *explicit and systematic instruction*. Students who experience persistent difficulties may benefit from clearly sequenced instruction, explicit modeling, guided practice, opportunities for independent practice, and frequent feedback. Evidence from intervention research has identified explicit instructional approaches among strategies that can support students experiencing mathematics difficulties.

A second principle is the use of *multiple representations and concrete or visual supports*. Mathematical concepts may be difficult when students are required to move directly from concrete experiences to abstract symbolic representations. Concrete materials, visual models, diagrams, number representations, and structured examples can provide additional pathways for connecting mathematical concepts with symbolic procedures.

A third principle is *differentiated and individualized instruction*. Because students with mathematics difficulties do not constitute a homogeneous group, teachers may need to adjust the pace, representation, level of scaffolding, amount of guided practice, or complexity of mathematical tasks according to students’ needs.

A fourth principle concerns *problem-solving and conceptual support*. Research syntheses have identified word-problem solving, fractions, and schema-based instruction as important areas of intervention research (Nelson et al., 2022). In middle-school settings, intervention research has demonstrated the potential benefits of targeted instruction across different mathematical content areas (Powell et al., 2021).

A fifth principle is *technology-supported mathematics instruction*. Digital interventions may provide opportunities for repeated practice, immediate feedback, visual representations, and adaptive learning experiences. A recent systematic review and meta-analysis of digital mathematics interventions for K–12 students with mathematical learning disabilities found a positive overall effect on mathematics learning outcomes, although the authors also emphasized the relatively limited empirical evidence base and the need for continued research (Alam et al., 2025).

Finally, *peer-assisted and collaborative learning* may provide opportunities for students to receive support while participating in ordinary classroom activities. Such approaches are particularly relevant to inclusive education because support does not necessarily require students experiencing mathematics difficulties to be separated from their peers.

Taken together, these perspectives suggest that effective instructional responses should be viewed as a coordinated set of practices rather than isolated techniques. The present study therefore examines teachers’ reported use of concrete materials, individualized instruction, technology-supported activities, and peer-assisted learning as manifestations of their broader instructional responses to students experiencing mathematics learning difficulties.

### 2.5 Significance and contribution of the present study

The existing literature provides substantial evidence concerning the cognitive characteristics of mathematics learning difficulties and the effectiveness of specific mathematics interventions. However, important gaps remain. First, the literature contains substantial conceptual and operational variation in the identification of mathematics learning difficulties (Nelson et al., 2022). Second, intervention research has predominantly focused on student outcomes, while teachers’ interpretations of students’ difficulties and their perceived readiness to address these difficulties have received comparatively less qualitative attention. Third, research on inclusive mathematics education has increasingly emphasized teachers’ beliefs and self-efficacy, but these dimensions are not always examined together with teachers’ concrete descriptions of students’ mathematical difficulties and instructional responses (Nührenbörger et al., 2025; Scherer and Bertram, 2024).

The present study addresses this gap by bringing these dimensions together within a qualitative framework. Rather than clinically identifying students with MLD, the study investigates how practicing middle-school mathematics teachers perceive and describe students whom they experience as having substantial and persistent difficulties in mathematics.

The study makes three main contributions.

*Theoretically*, it brings together a multidimensional conceptualization of mathematics learning difficulties with the literature on teacher beliefs, self-efficacy, and inclusive mathematics teaching. The study does not test a causal theoretical model; rather, these perspectives provide a conceptual lens for interpreting teachers’ accounts.

*Empirically*, the study provides qualitative evidence concerning how middle-school mathematics teachers identify mathematical and affective difficulties, what instructional practices they report using, and how they perceive their own competence and professional preparation.

*Practically*, the findings may inform mathematics teacher education and professional development by identifying areas in which teachers perceive a need for additional knowledge, training, instructional resources, and professional support.

The Turkish context also warrants attention because inclusive mathematics teaching is shaped by national educational structures, teacher preparation systems, curriculum expectations, school resources, and professional support opportunities. Evidence generated in the Turkish middle-school context can therefore provide context-sensitive insight into how mathematics teachers interpret and respond to students experiencing mathematics learning difficulties.

Accordingly, the present study focuses on the following questions:

1. How do mathematics teachers identify and assess students’ difficulties with mathematics?
2. Which teaching methods do teachers prefer to support students who struggle with mathematics?
3. What are the teachers’ views regarding the topics that students struggling with mathematics encounter?
4. Do teachers consider themselves competent in implementing the necessary educational practices for students who struggle with mathematics?

By examining teachers’ beliefs, perceptions, and instructional responses, this study aims to contribute to a deeper understanding of the psychological and pedagogical processes underlying mathematics learning support and provide implications for teacher education and inclusive mathematics practices.

## 3 Conceptual model

The conceptual model of teachers’ beliefs and instructional approaches towards students with mathematics learning difficulties is given in Figure 1.

**Figure 1:**
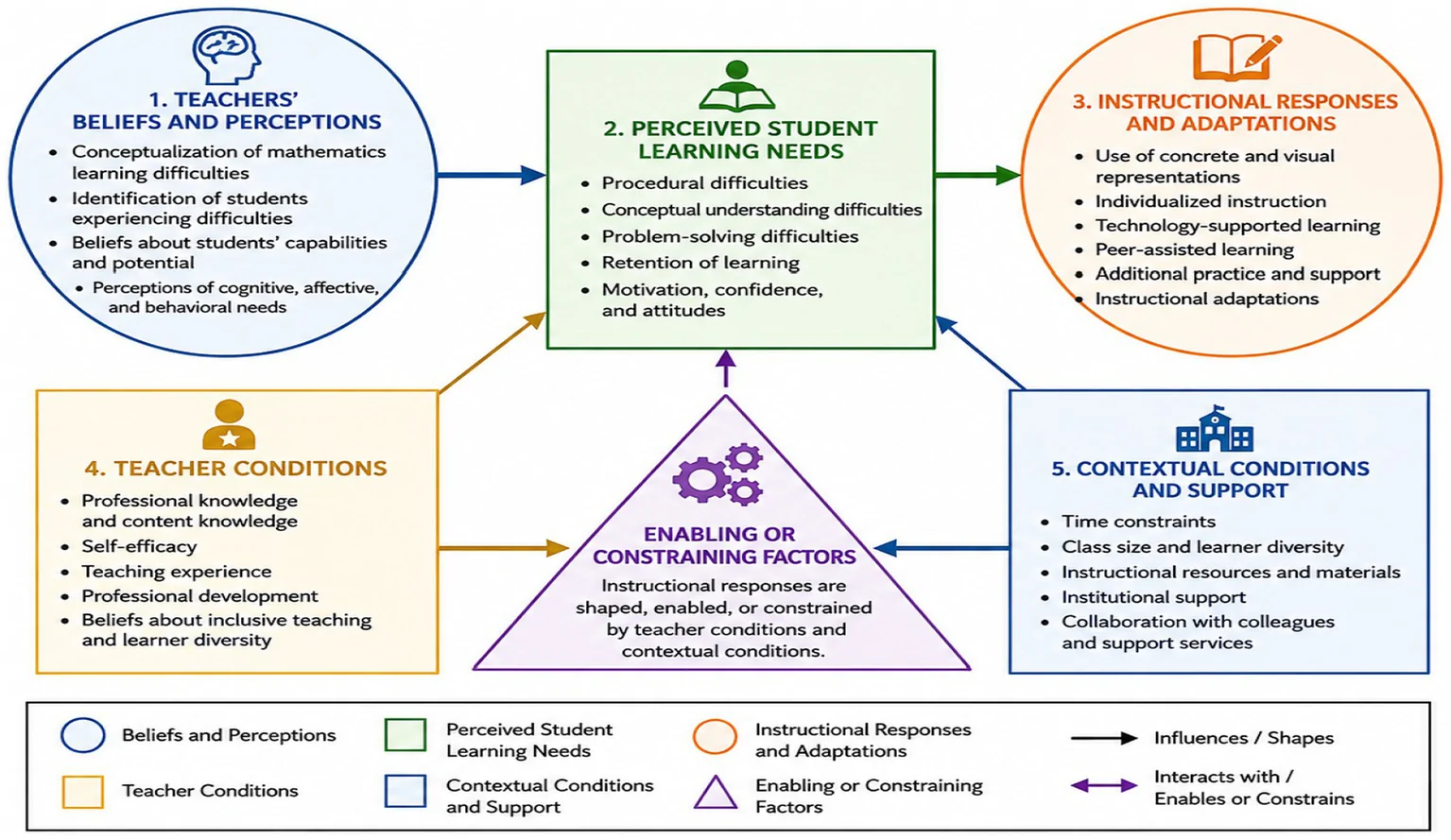
Conceptual model of mathematics teachers’ beliefs and instructional responses toward students with mathematics learning difficulties from an inclusive mathematics education perspective. This model illustrates how teachers’ beliefs and perceptions, perceived student learning needs, instructional responses and adaptions, teacher conditions, and contextual conditions and support interact with one another. Instructional responses are shaped, enabled, or constrained by teacher conditions and contextual conditions. The model represents an analytical framework for interpreting qualitative data and does not imply a causal relationship.

Figure 1 illustrates the analytical logic underlying the present study. The model begins with teachers’ beliefs and perceptions because teachers’ interpretations of mathematics learning difficulties influence how they recognize and make sense of students’ learning needs. However, these perceptions are not viewed as fixed determinants of instructional practice. Rather, they interact with teachers’ professional knowledge, perceived self-efficacy, previous experiences, and the contextual conditions in which instruction takes place. The second component of the model concerns teachers’ perceptions of students’ learning needs. The findings suggest that teachers identify difficulties across procedural, conceptual, problem-solving, and affective dimensions. These perceptions subsequently inform the instructional responses that teachers report using, including concrete and visual representations, individualized instruction, technology-supported activities, peer assistance, and additional learning opportunities. Thus, instructional adaptation is conceptualized as a process through which teachers attempt to respond to perceived barriers to students’ mathematical participation and understanding. Importantly, the model does not position instructional responses as solely dependent on teachers’ individual beliefs or competencies. Teachers’ professional knowledge and self-efficacy, together with contextual conditions such as time, class heterogeneity, instructional resources, professional development opportunities, and access to collaborative support, may enable or constrain their capacity to implement inclusive practices. The model therefore adopts a person–practice–context perspective, emphasizing that inclusive mathematics teaching is produced through the interaction between what teachers believe, what they perceive students need, what they are able to do instructionally, and the conditions under which they work. The conceptual model consequently provides an integrative lens for interpreting the qualitative findings. Rather than treating mathematics learning difficulties as characteristics located exclusively within students, it directs attention to the reciprocal relationship between students’ perceived learning needs, teachers’ interpretations and instructional responses, and the professional and institutional conditions surrounding teaching. In this respect, the model supports the central premise of the study that more inclusive mathematics education requires not only teachers’ willingness to adapt instruction but also sufficient professional knowledge, self-efficacy, resources, and collaborative support to make such adaptation sustainable.

## 4 Method

This study adopted a qualitative case study design to examine mathematics teachers’ experiences, perceptions, and instructional responses regarding students experiencing mathematics learning difficulties (MLD) within an inclusive education context. A qualitative approach was appropriate because the study sought to understand how teachers interpret MLD, how they perceive their roles and competencies, and how they describe their instructional responses and challenges in their natural educational settings (Creswell and Poth, 2018; Patton, 2015).

A case study design was selected because it enables an in-depth and context-sensitive examination of a complex educational phenomenon within its real-life setting (Yin, 2018). In the present study, the case was defined as mathematics teachers’ experiences and perceptions of supporting students experiencing MLD in inclusive middle school mathematics classrooms. The case was bounded by three interrelated dimensions: **(**a) participants, comprising middle school mathematics teachers with experience teaching students whom they perceived as experiencing persistent difficulties in mathematics learning; (b) context, comprising inclusive mathematics classrooms in middle schools; and (c) phenomenon, comprising teachers’ perceptions of MLD, perceived professional competencies, instructional responses, challenges, and support needs.

The case study design was considered more appropriate than a phenomenological design because the purpose of the study was not to identify the essential structure of teachers’ lived experiences. Rather, the study aimed to examine a multifaceted educational phenomenon by considering the relationships among teachers’ perceptions, instructional responses, professional competencies, and contextual challenges. Similarly, the study was not intended as a purely descriptive account of teachers’ experiences; instead, it sought to interpret these experiences within the broader context of inclusive mathematics education.

The study was not designed to establish causal relationships or statistical generalizations. Instead, it aimed to generate rich and context-dependent insights into how mathematics teachers understand and respond to students experiencing MLD. The primary source of data was semi-structured interviews with participating teachers. Although classroom observations and documents, such as instructional plans, student progress records, and supplementary teaching materials, were consulted during the research process, these sources were not systematically collected, coded, or included in the formal data analysis. Therefore, they were used only to enhance the researchers’ contextual understanding of the case and were not treated as independent data sources. Accordingly, the study does not claim methodological or data-source triangulation.

Importantly, the study focuses on teachers’ perceptions and professional experiences rather than formal diagnoses of mathematics learning disabilities. The identification of students experiencing MLD was based on teachers’ classroom observations and professional judgments. This distinction is important because teachers’ interpretations may reflect their professional knowledge, expectations, and classroom experiences and should not be regarded as clinical or diagnostic classifications of students (Leymun et al., 2017). Consequently, the findings are interpreted as representing teachers’ understandings and instructional responses to perceived mathematics learning difficulties rather than objectively diagnosed learning disabilities.

### 4.1 Participants and sampling

This study was conducted with 20 mathematics teachers working in middle schools in Turkey’s Eastern Anatolia Region. Participants were selected using purposive sampling, as there is a need for teachers with experience working with students who struggle with mathematics. In line with the study’s objective, a purposive sampling strategy based on criteria sampling was employed (Patton, 2015). Criteria sampling ensures that all participants meet specific conditions related to the research topic. The selection criteria were as follows: (1) being a middle school mathematics teacher, (2) having taught students who consistently struggle with mathematics, and (3) voluntarily agreeing to participate in the study. The sample size was determined based on qualitative research principles rather than statistical power analysis. Specifically, participant selection was based on the relevance and richness of the information provided by the participants, as well as the extent to which the interviews addressed the research questions. In other words, the sample size was determined based on data saturation. The data collection process continued until no new themes or categories emerged from the interviews, indicating that sufficient depth had been achieved (Creswell and Poth, 2018). Accordingly, the final sample consisted of 20 mathematics teachers. The purpose of the sample was not to provide a statistical representation of mathematics teachers, but rather to offer rich, context-based insights into the teachers’ experiences and perceptions. Consequently, the data obtained from the 20 teachers was deemed sufficient to comprehensively address the research questions. In terms of demographic characteristics, 40% of the participants (*n* = 8) were female and 60% (*n* = 12) were male. In terms of educational background, 85% held a bachelor’s degree, while 15% held a master’s degree. Additionally, 35% of the participants had 5–10 years of professional experience, 55% had 11–20 years, and 10% had 21–30 years.

### 4.2 Data collection tool

The data were collected through a semi-structured interview form developed by the researcher. The interview form consisted of two sections: the first included questions regarding participants’ demographic characteristics, and the second included open-ended questions focusing on teachers’ experiences, perceptions, and instructional practices related to students with mathematics learning difficulties. Semi-structured interviews were preferred because they provide flexibility for probing deeper into participants’ responses while maintaining a consistent framework across interviews (Creswell and Poth, 2018). This approach enabled the researcher to ask follow-up questions and obtain more detailed information when necessary. During the development of the interview form, a comprehensive review of the relevant literature was conducted. Based on this review, the initial interview questions were prepared in line with the purpose and direction of the study. The interview questions focused on four key areas: 1. Teachers’ perceptions regarding students experiencing mathematics learning difficulties; 2. Teachers’ observations concerning students’ mathematical learning needs; 3. Instructional practices used to support students; and 4. Teachers’ perceived competence, preparedness, and professional development needs. Thus, the direct relationship between the interview questions and the research questions was established. Again, to ensure content validity, expert opinions were sought from a qualitative research specialist, a mathematics education expert, and an experienced mathematics teacher holding a doctoral degree. Revisions were made in line with their feedback. A pilot study was conducted with two mathematics teachers to test the clarity and relevance of the questions. Based on the feedback obtained from the pilot application, necessary adjustments were made, and the interview form was finalized.

### 4.3 Data collection

Data were collected through individual semi-structured interviews with the participating mathematics teachers. This format was selected because it provided a consistent structure across participants while allowing the researchers to explore individual experiences, examples, and explanations in greater depth. The interview protocol was developed based on the relevant literature on mathematics learning difficulties, inclusive education, mathematics teaching practices, and teacher professional competencies. The questions addressed four main areas: (1) teachers’ conceptualizations and identification of mathematics learning difficulties; (2) their beliefs and perceptions regarding students experiencing these difficulties; (3) the instructional strategies and adaptations they used to support these students; and (4) the challenges, professional competencies, and support needs associated with inclusive mathematics teaching. Follow-up and probing questions were used when participants’ responses required clarification, elaboration, or concrete examples. This approach enabled teachers to describe their experiences in their own words while ensuring that the main topics relevant to the research questions were addressed consistently across interviews. The interviews were conducted in a setting that allowed participants to express their experiences freely and confidentially. With participants’ consent, all interviews were audio-recorded and subsequently transcribed verbatim for analysis. During the interviews, the researchers also recorded key points and contextual details to preserve the meaning and consistency of participants’ accounts. To enhance the researchers’ contextual understanding of the instructional setting, classroom observations and document reviews were also conducted during the research process. Specifically, three 40-min mathematics lessons were observed to gain contextual insight into teachers’ instructional practices with students experiencing mathematics learning difficulties. In addition, relevant instructional materials, lesson plans, classroom notes, remedial activities, student progress records, and Supplementary materials used to support these students were reviewed. However, these observations and documents were not systematically coded or analyzed as independent data sources. Rather, they were used to familiarize the researchers with the educational context and to inform the interpretation of the interview data. Accordingly, the formal findings reported in this study are based primarily on the semi-structured interview data, and no claim of data-source triangulation is made. Before data collection, participants were informed about the purpose and procedures of the study, the voluntary nature of participation, and the confidentiality of the information they provided. Written informed consent was obtained from all participants.

### 4.4 Data analysis

Data were analyzed using Braun and Clarke’s (2006) thematic analysis framework. The analysis involved familiarization with the interview transcripts, generation of initial codes, identification of candidate themes, review and refinement of themes, definition and naming of themes, and production of the final thematic account. The analysis process followed an inductive approach in which themes and categories were generated from participants’ responses rather than being predetermined. First, all interviews were transcribed verbatim and carefully reviewed several times to ensure familiarity with the data. During the initial coding phase, meaningful statements related to teachers’ perceptions, instructional practices, and challenges regarding students with mathematics learning difficulties were identified and assigned initial codes. Following the initial coding process, related codes were compared, grouped, and organized into broader categories. Subsequently, these categories were examined to develop overarching themes representing the common patterns across participants’ experiences. To ensure consistency and coherence in the analysis process, the themes were reviewed repeatedly and refined by considering their internal consistency and relationship with the research questions. The final themes and sub-themes were determined through continuous comparison of coded data and the original interview transcripts. To enhance the credibility of the findings, several strategies were employed. First, the researcher maintained a clear record of the coding process and analytical decisions throughout the study. Second, expert review was obtained from a qualitative research specialist who examined the coding framework and provided feedback regarding the appropriateness of the themes. Third, direct quotations from participants were included in the findings section to demonstrate the connection between participants’ statements and the interpreted themes. The reliability of the qualitative analysis was addressed through consistency checks during the coding process. Following Miles et al. (2014) recommendation, agreement between the researcher’s coding and expert evaluation was examined. Any disagreements regarding codes or themes were discussed and resolved through consensus. These procedures contributed to the credibility, dependability, and confirmability of the findings. Moreever, nitel veri analizi sürecinde, katılımcıların ifadeleri birden fazla anlam birimini temsil ettiğinde birden fazla koda atanmasına izin verildi. Bu nedenle, bulgular tablolarında sunulan toplam frekanslar katılımcı sayısını (*n* = 20) aşabilir.

### 4.5 Validity and reliability

It is noted that in qualitative research, credibility should take precedence over validity and reliability (Houser, 2015). Credibility is addressed within the framework of the “gold standards” proposed by Lincoln and Guba (1985) under four main headings: 1. Credibility: Systematically establishing the connections between data, codes, themes, and research questions; 2. Transferability: Providing a detailed description of the participants, the educational setting, and the research context; 3. Consistency/Dependability: Clearly and transparently describing the data collection and analysis processes; 4. Confirmability: Clearly linking the emerging interpretations to participant statements and the obtained data. In this study, the data collected from participants were analyzed using content analysis, one of the qualitative data analysis methods. Content analysis is a process that involves collecting and interpreting similar information around specific themes and topics (Yıldırım and Şimşek, 2018). In qualitative research, validity refers to presenting the subject of the study in an unbiased manner, covering all its aspects without incorporating the researcher’s personal views. It also encompasses verifying the reliability and acceptability of the methods used to collect data objectively. In qualitative research, internal validity is ensured through credibility. Credibility provides information on how well the results obtained in the study align with and are connected to reality (Arastaman et al., 2018). The most important elements of persuasiveness can be listed as the length of the interviews, participant validation, and expert evaluation. Before beginning the research, a pilot study was conducted to assess the appropriateness and functionality of the interview questions. Following the pilot study, the interview questions were finalized. After the pilot study, the institutions where the interviews would be conducted were identified, and interviews were conducted with a total of 8 female and 12 male teachers. Prior to the interview, the “Informed Consent Form” was read and approved. During the interview, participant validation was ensured after each question.

In qualitative research, reliability is considered a key factor in enhancing the persuasiveness of the study. Furthermore, the reliability of qualitative research can be defined as the reproducibility of the results obtained. Reliability in qualitative research increases when similar studies conducted by different researchers yield the same results (Yıldırım and Şimşek, 2018). In this context, the records related to the research data were reviewed by two experts in the field and transcribed into text. At the conclusion of the analysis, it was observed that there was an 82.0% consistency in the data among the experts. This percentage represents coding reliability in qualitative research, according to Miles and Huberman (1994). In the study, the percentage of participants’ responses to the questions was calculated, and the results were presented in tables. Before the study began, teachers were informed that their personal information would remain confidential and that the interviews would be used solely for research purposes. The teachers’ identities were kept confidential and coded as (T1, T2, …, 20). To ensure consistency in qualitative data analysis, two researchers independently coded the interview transcripts. After comparing the coding results, disagreements were discussed until a consensus was reached. The reliability of the coding process was calculated using Miles and Huberman’s (1994) formula: Reliability = Agreement/(Agreement + Disagreement) × 100. The agreement coefficient obtained in this study indicated an acceptable level of consistency between the researchers.

### 4.6 Ethical considerations

The study was conducted in accordance with established ethical principles for research involving human participants. Ethical approval was obtained from the relevant institutional ethics committee before data collection. Participation was voluntary, and all participants were informed about the purpose of the study, the confidentiality of their responses, and their right to withdraw from the study without consequence. Written informed consent was obtained from all participating teachers. Because the study involved adult mathematics teachers and did not collect data directly from students, students were not treated as research participants. The study focused exclusively on teachers’ professional experiences and perceptions regarding students experiencing mathematics learning difficulties. All identifying information was removed from the transcripts and reports, and participant codes were used throughout the manuscript to protect confidentiality.

## 5 Results

This section presents the teachers’ views regarding the questions addressed in the study, along with their original statements that align with these views.

### 5.1 Teachers’ views regarding the first question addressed in the study “How do mathematics teachers identify and evaluate the difficulties students experience with mathematics?”

**Table 1:** Teachers’ views on identifying math learning difficulties.

Theme subtheme code *f*	
Mathematics learning difficulty	Difficulty in learning 12 Process related difficulties difficulties encountered in Mathematical operations 8 Forget what you learn fast 5 Persistence of failure 3
	Lagging behind peers 10 Teacher perception teacher awareness 5
	Emotional effects prejudice against mathematics 11

As shown in Table 1, teachers’ identification of students with mathematics learning difficulties is primarily based on observable and persistent difficulties in the learning process. Within the “process-related difficulties” subcategory, learning difficulties (*f* = 12) and difficulties performing mathematical operations (*f* = 8) were the most frequently reported indicators. Another notable indicator was the forgetting of previously learned content (*f* = 5). Teachers interpreted students’ inability to retain previously taught information and apply it later as evidence that learning had not yet become sufficiently consolidated. Similarly, persistent failure (*f* = 3) was identified as a significant sign of ongoing difficulty. The “teacher perception” subcategory further highlights the importance of classroom observation in this assessment process. Falling behind peers (*f* = 10) was one of the most frequently reported indicators and showed that teachers generally interpreted students’ difficulties in relative terms by comparing them to their classmates’ mathematical progress. Teacher awareness (*f* = 5) similarly highlights the role of teachers’ accumulated classroom experience in identifying patterns of difficulty. The “emotional effects” subcategory adds an additional dimension to the teachers’ comments. Prejudice or negative attitudes toward mathematics (*f* = 11) were frequently identified as a significant indicator. The participants’ accounts demonstrate this interaction between observable performance, perseverance, and emotional factors. For example, T-1 highlighted the relatively high number of students struggling with the subject and the significant effort required to help them reach their peers’ level:

*“The number of students in my class who struggle with math is quite high. The percentage of students who score low on my tests is also quite high. We need to make a concerted effort to help these students reach the same level as their peers.” (T-1).*

This statement shows that teachers’ perception of difficulty is closely related to students’ assessment performance and their standing relative to their peers. More importantly, this statement reflects a performance-oriented interpretation of difficulty, in which success is defined, in part, by reaching the same level as classmates. T-5 offered a more complex narrative highlighting the inconsistency in students’ learning despite repeated instructional efforts:

*“When working with students who struggle in class, I always think: If I work hard, I’ll succeed. If you put in enough effort, you’ll definitely understand, but sometimes I feel that no matter what we do, the children just cannot grasp the topic. There are also moments when I think: Great, they understood this time. But after a while, I see them struggling with the same question I thought they had already understood.” (T-5).*

This account shows that teachers distinguish between short-term task success and lasting mathematical learning. The repetition of the same challenge following the apparent improvement led the teacher to reinterpret the initial success as insufficient evidence of lasting understanding. Similarly, T-11 described how students’ difficulties persisted despite repeated explanations:

*“I try to explain or solve problems that some students cannot solve or topics they cannot understand over and over again, but the student responds, ‘Teacher, no matter what you do, I cannot understand math; my mind just cannot grasp it.’” (T-11).*

This statement points to a potential tension between teachers’ efforts to provide additional explanations and students’ ongoing difficulties in understanding mathematical content. What is important is that repeated explanation appears to be a dominant initial response; this suggests that some teachers may interpret learning difficulties primarily as a need for more explanation or practice, rather than as a signal to reevaluate their teaching approach, representations, task design, or learning environment. However, T-7 emphasized that some students demonstrate significant effort despite limited academic progress:

*“I know that students with learning difficulties put in a lot of effort; most of them participate well in class and prepare diligently for exams, but they generally make very little progress on math exams.” (T-7).*

This statement complicates a purely deficit-focused interpretation. It demonstrates that teachers acknowledge a distinction between effort and achievement, recognizing that low mathematical performance does not necessarily reflect low motivation or insufficient effort.

### 5.2 Teachers’ views regarding the second research question “Which teaching methods do teachers prefer to support students who struggle with mathematics?”

**Table 2:** Teachers’ views on educational methods applied to mathematics learning difficulties.

Theme subtheme code *f*	
Learning Teaching Practices	Teaching by concretizing 18 Teaching methods and teaching in one-to-one lessons 14 Techniques teaching using technology 12 Cascading method 5
	Peer support 7 Supportive strategies providing opportunities to succeed 5 Additional homework support 5 Preparing separate materials 4
	Learning environment creating a comfortable and natural 10 Configuration creating a U seating arrangement 6 Blocking attention-grabbing elements 4 Preventing competition 2
	Psychosocial supports making everyone equally responsible 2 No peer differentiation 2

As shown in Table 2, teachers reported using a range of instructional and classroom-management strategies to support students experiencing mathematics learning difficulties. The most frequently reported practices were teaching through concrete examples (*f* = 18), one-to-one instruction (*f* = 14), and technology-supported instruction (*f* = 12). The prominence of these practices suggests that teachers tend to respond to perceived learning difficulties by increasing the concreteness of mathematical content, providing additional individualized support, and using alternative instructional resources.

The frequent use of concrete examples is particularly noteworthy. Teachers appeared to use familiar and everyday contexts to reduce the abstractness of mathematical concepts and make mathematical relationships more accessible. T-2, for example, described connecting mathematical problems to students’ everyday experiences:

*“In mathematics lessons, I usually illustrate the topics with examples from daily life. In order for students to solve mathematical problems, I often visit the bakery to buy bread. For example, suppose a student has 80 Turkish Liras. The price of a loaf of bread is 10 Turkish Liras. If this student buys three loaves of bread, I question how much money he has to give to the baker and how much money he has left. With such examples, I try to integrate students’ shopping experiences with mathematics.” (T-2).*

This account illustrates how teachers attempt to mediate mathematical abstraction through familiar experiences. The second most frequently reported strategy was one-to-one instruction (*f* = 14). Teachers described providing additional individual support when whole-class instruction was perceived as insufficient. T-9 explained:

*“In order for students with learning difficulties to perform better, I mostly work with them one-on-one after regular class hours. However, I give them make-up lessons three evenings a week. I review the additional lessons I give and provide feedback to the students.” (T-9).*

This response demonstrates teachers’ willingness to invest additional time and effort in addressing individual learning needs. Technology-supported instruction (*f* = 12) was another frequently reported response. Other strategies were reported less frequently, including peer support (*f* = 7), creating opportunities for success (*f* = 5), additional homework (*f* = 5), and preparing special materials (*f* = 4). Although these practices occurred less frequently in the dataset, they are analytically important because they indicate that teachers’ responses were not limited to additional explanation or repetition. The role of classroom organization was also evident. Teachers reported providing a comfortable and natural classroom environment (*f* = 10) and using a U-shaped seating arrangement (*f* = 6). In addition, reducing attention-grabbing elements (*f* = 4) was mentioned as a strategy for minimizing distractions. These practices suggest that some teachers interpreted students’ difficulties not solely in terms of mathematical knowledge but also in relation to the conditions under which learning takes place. The physical and social organization of the classroom was therefore considered part of the instructional response. T-12 explicitly acknowledged the complexity of managing a classroom characterized by different levels of mathematical ability:

*“There are a few students in my class who struggle in math. I know the difficulties of managing a class with different abilities, how each student affects the others and how it puts extra pressure on the teachers. However, I try not to make the students feel this situation.” (T-12).*

This statement is particularly significant from an inclusive education perspective. The teacher recognizes learner diversity as a normal characteristic of the classroom while simultaneously attempting to prevent students experiencing difficulties from being stigmatized or differentiated negatively. Thus, the instructional response extends beyond academic remediation to include the protection of students’ social participation and classroom belonging. Similarly, T-18 described the use of mathematically proficient peers as a form of peer-assisted support:

*“Sometimes I make use of students who are talented in mathematics. In this way, students comprehend each other better and do not hesitate to ask about the subjects they do not understand.” (T-18).*

Although these practices occurred less frequently in the dataset, they are analytically important because they indicate that teachers’ responses were not limited to additional explanation or repetition.

### 5.3 Teachers’ views regarding the third research question “What are the teachers’ views regarding the topics that students struggling with mathematics encounter?”

**Table 3:** Teachers’ views on the topics that students who struggle with math have difficulty with.

Theme	Subtheme	Code	*f*
Mathematics subjects	Numbers and operations	Addition, subtraction, multiplication and division Problem solving Recognizing Numbers and Symbols Fractions Step values Rounding to tenths	19 18 12 6 4 2 12 6 2
	Units of measurement	Hours Units of measurement and conversion Coins	
	Geometry	Geometric shapes Three-dimensional objects Size and orientation of objects	5 2 2
	Statistical data	Reading and interpreting data from tables	3

The distribution presented in Table 3 indicates that teachers perceived mathematics learning difficulties across several content domains, with the most frequently reported difficulties concentrated in basic numerical operations and problem solving. Within the numbers and operations category, difficulties with addition, subtraction, multiplication, and division (*f* = 19) and problem solving (*f* = 18) were the most frequently reported. In contrast, rounding to tens was reported as the least problematic topic within this category. The high frequency of problem-solving difficulties (*f* = 18) provides a further analytical dimension. A similar pattern was evident in the measurement domain. Clock-related tasks (*f* = 12) were the most frequently reported difficulty, whereas coins (*f* = 2) were reported less frequently. The relatively high frequency of difficulties with clocks may reflect the multiple representations involved in time-related tasks, including numerical notation, units, sequences, and relationships between units of time. In geometry, geometric shapes (*f* = 5) were the most frequently reported difficulty, while dimensions and orientations of objects (*f* = 2) were reported less frequently. The statistical data category was mentioned less frequently. Teachers reported some difficulty with reading and interpreting data presented in tables (*f* = 3). Although this was not among the most frequently identified difficulties, it is analytically relevant because data interpretation requires students to coordinate visual information with numerical and relational reasoning. The teachers’ accounts provide further insight into how these content-specific difficulties were interpreted and addressed. T-15 described the use of visual and concrete materials when teaching geometry:

*“I see that students who have difficulty in learning while explaining topics related to geometry have difficulty in perceiving geometric shapes, so I bring related materials to the lesson so that students can see and recognize the shapes visually so that they can perceive geometric shapes more easily.” (T-15).*

This account illustrates an important relationship between teachers’ perceptions of students’ difficulties and their instructional responses. The teacher interpreted the difficulty not simply as an inability to learn geometry but as a problem involving the perception and recognition of geometric forms. T-20 similarly emphasized the importance of connecting mathematical concepts with everyday experiences:

*“Considering the presence of students with learning difficulties in the classroom, I always make use of concrete objects and materials while teaching mathematics lessons. For example, when I teach the topic about money, I present the currencies used in daily shopping to the students as visual materials and make my explanations. In this way, students comprehend the topics better. For this reason, we try to make the subjects as concrete as we can.” (T-20).*

The account suggests that teachers consciously attempt to reduce the representational demands of mathematical content by connecting abstract concepts with tangible objects and familiar situations. A similar approach was reported by T-16 in relation to basic operations:

*“I perform addition and subtraction operations with concrete objects; in this way, students who have difficulty in mathematics can perform operations by observing unities and tens. This approach offers both a concrete and fun method.” (T-16).*

Here, concrete materials are used to make the underlying structure of addition and subtraction visible. The teacher’s reference to units and tens suggests an attempt to support students’ understanding of place value rather than relying solely on symbolic notation. Thus, the instructional response can be interpreted as an effort to bridge concrete, representational, and symbolic forms of mathematical knowledge. T-13 further emphasized the role of visual representations in problem solving:

*“After a mathematics lesson, I see that some students cannot comprehend the solution while solving the questions of that subject. However, when I show the solution with graphs and figures to the students who do not understand, I realize that I get a more effective result.” (T-13).*

This account is particularly relevant because it connects the frequently reported problem-solving difficulties with the use of alternative representations. The finding therefore reinforces the interpretation that some teachers perceive learning difficulties as partly related to the fit between instructional representation and students’ ways of accessing mathematical meaning.

### 5.4 The teachers’ views regarding the fourth research question “Do they consider themselves competent in implementing the necessary educational practices for students experiencing difficulties in mathematics?"

**Table 4:** Teachers’ competencies regarding students struggling with mathematics.

Theme	Subtheme	Code	f
Teachers’ perceived self-efficacy	Perceived limitations in inclusive mathematics teaching	Feeling insufficient due to limited expertise and experience	
		Need for specialized training on mathematics learning difficulties	15
	Instructional challenges	Difficulty selecting appropriate methods and techniques	
	Sources of perceived competence	Adapting instruction according to students’ levels	
		Using appropriate teaching methods and techniques	5
		Collaboration with guidance and research services	

Table 4 the analysis revealed that teachers’ perceptions of self-efficacy were not limited to a simple distinction between being competent or incompetent; rather, these perceptions were shaped by teachers’ professional knowledge, classroom experiences, instructional challenges, and access to institutional support. Most teachers (*f* = 15) expressed concerns regarding their ability to effectively support students with mathematics learning difficulties. Teachers with lower perceived self-efficacy mainly associated their concerns with insufficient knowledge and limited preparation for inclusive mathematics teaching. They emphasized that students with mathematics learning difficulties require specialized instructional approaches and that the absence of adequate training may lead to ineffective practices. As one teacher explained:

*“If we do not receive special training for students with learning difficulties, there is a risk of applying inappropriate methods and techniques to these students.” (T-17).*

Another teacher highlighted that supporting these students creates additional instructional responsibilities and requires continuous professional improvement:

*“It is clear that the presence of students who have difficulty in mathematics in the classroom definitely creates an additional burden for teachers. To put it bluntly, I don’t feel completely adequate because as teachers, we may also have shortcomings and there are definitely areas where we need to improve.” (T-14).*

On the other hand, a smaller group of teachers (*f* = 5) reported higher self-efficacy. These teachers emphasized the importance of adapting instruction according to students’ levels, using appropriate teaching strategies, and collaborating with professional support services. For example, one teacher stated:

*“Yes, I think I am adequate, because I try to teach the content in accordance with the level of the students by using appropriate methods and techniques, with preliminary education in this field, continuous cooperation with guidance and research center, psychological counseling.” (T-19).*

The findings indicate that teachers’ self-efficacy perceptions were primarily associated with their professional knowledge and experience in supporting students with mathematics learning difficulties.

## 6 Discussion

The aim of this study was to explore mathematics teachers’ perceptions, instructional practices, and challenges regarding students who experience difficulties in learning mathematics. The findings provide insights into how teachers identify mathematics learning difficulties, which instructional strategies they employ, and what types of professional support they need. The results are discussed in relation to previous research on mathematics learning difficulties, inclusive mathematics education, and teacher professional competence.

### 6.1 Teachers’ views on methods of identifying learning difficulties in mathematics

The findings indicate that teachers primarily identify mathematics learning difficulties through observable academic indicators, including difficulties in mathematical operations, insufficient basic skills, low achievement levels, and students’ inability to keep pace with their peers. This suggests that teachers tend to interpret mathematics learning difficulties mainly through students’ classroom performance and academic outcomes. This finding is consistent with previous research indicating that teachers often rely on observable behaviors and achievement-related indicators when recognizing students who experience difficulties in mathematics. Students with mathematics learning difficulties commonly demonstrate persistent challenges in numerical understanding, calculation accuracy, mathematical reasoning, and problem-solving processes (Szűcs and Goswami, 2013). Therefore, teachers’ emphasis on basic mathematical skills reflects important characteristics associated with mathematics learning difficulties. However, the findings also suggest that teachers’ interpretations may sometimes remain limited to performance-based indicators. Although academic performance provides valuable information, identifying mathematics learning difficulties requires consideration of multiple dimensions, including cognitive processes, affective factors, instructional experiences, and learning environments (Nelson et al., 2022). A narrow focus on achievement outcomes may lead teachers to perceive difficulties as individual student deficits rather than considering the interaction between student characteristics and instructional contexts. Another important finding is that teachers associated mathematics learning difficulties with students’ low motivation, negative attitudes toward mathematics, and reduced confidence. Previous research has demonstrated that affective variables such as mathematics anxiety, self-efficacy, and attitudes toward mathematics are closely related to students’ mathematical engagement and achievement (Hamukwaya and Haser, 2021). However, these affective characteristics should be interpreted carefully because they may represent consequences of repeated failure experiences rather than direct causes of learning difficulties. Therefore, teachers’ awareness of the reciprocal relationship between academic difficulties and emotional factors is essential for developing effective support strategies. The findings also revealed that some teachers considered students’ performance compared with their peers as an important indicator of mathematics learning difficulties. While classroom comparisons may help teachers recognize students who need additional support, inclusive education principles emphasize the importance of avoiding deficit-oriented perspectives and maintaining high expectations for all learners. Effective mathematics instruction for students with learning difficulties requires teachers to recognize individual learning needs while providing appropriate opportunities for participation and success (Joswick et al., 2023).

The results of this study reveal that mathematics teachers view mathematics learning difficulties (MLD) as a multifaceted phenomenon encompassing cognitive, emotional, and instructional dimensions. While previous studies have generally focused on cognitive explanations such as deficiencies in numerical operations, lack of conceptual understanding, and weaknesses in procedural skills (Dowker, 2019), the current data expand this approach by showing that teachers also recognize emotional factors such as math anxiety, low motivation, and underdeveloped self-confidence as significant components of students’ learning difficulties. This finding appears consistent with modern perspectives on mathematics education, which argue that individuals’ mathematical development is shaped by the interactions among cognitive resources, emotional experiences, and learning environments (Ramirez et al., 2018). From this perspective, it should be recognized that difficulties in learning mathematics do not stem solely from the characteristics of individual students, but are also challenges rooted in the relationships between learners and teaching practices. This study contributes to this discussion by demonstrating how teachers’ professional perspectives influence the identification and management of learning difficulties in the classroom. It can be argued that teachers who understand the emotional and contextual dimensions of learning difficulties are more likely to adopt supportive and adaptive teaching methods rather than attributing these difficulties solely to students’ abilities.

Overall, the findings suggest that teachers possess practical awareness of several indicators associated with mathematics learning difficulties; however, they may benefit from professional development opportunities that support a more comprehensive understanding of these difficulties. Such programs should address not only academic indicators but also cognitive, emotional, and contextual factors influencing students’ mathematical development.

### 6.2 Teachers’ views on teaching methods applied for mathematics learning difficulties

The findings demonstrate that teachers employ various instructional strategies to support students experiencing mathematics learning difficulties, including the use of concrete materials, individualized instruction, technology-supported learning activities, and peer support. This diversity of strategies indicates that teachers recognize the importance of adapting instruction according to students’ individual needs. The frequent use of concrete materials reported by teachers is consistent with previous research emphasizing the importance of multiple representations in mathematics learning. Students with mathematics learning difficulties often experience challenges in understanding abstract mathematical concepts; therefore, concrete and visual representations can support conceptual understanding by connecting abstract ideas with meaningful experiences (Fuchs et al., 2012; Stevens et al., 2017). The teachers’ preference for concrete materials in the current study suggests an awareness of the need to make mathematical concepts more accessible. Teachers also frequently reported using individualized support practices. This finding aligns with research showing that students with mathematics learning difficulties benefit from systematic and targeted instructional interventions designed according to their specific learning needs (Lee et al., 2020). Individualized instruction allows teachers to adjust the pace, provide additional explanations, and offer opportunities for repeated practice. However, previous studies also emphasize that individualized support should be integrated with classroom-based differentiated instruction rather than being considered as the only solution (Nelson et al., 2022). Technology-supported instruction was another strategy identified by teachers. Previous research indicates that digital learning environments can support students with mathematics learning difficulties by providing immediate feedback, repeated practice opportunities, and adaptive learning experiences (Kiru et al., 2018; Ok et al., 2019; Re et al., 2020). Nevertheless, the effectiveness of technology depends not only on the availability of digital tools but also on teachers’ pedagogical decisions regarding how these tools are integrated into instruction. Therefore, technology should be considered as a supportive component of evidence-based teaching rather than a substitute for effective pedagogy. The findings also indicate that teachers use peer support and collaborative learning activities. Collaborative approaches may contribute to both academic and social development by increasing students’ engagement and participation in mathematics classrooms. However, successful implementation requires careful planning and teacher guidance to ensure that students with learning difficulties actively participate rather than remain passive recipients of support.

However, the findings also reveal a significant tension between teachers’ positive attitudes toward inclusive education and their perceived competence in implementing systematic approaches. Similarly, previous research has shown that while teachers generally support the principles of inclusive education, they struggle to translate these principles into classroom practice due to limited preparation, insufficient professional development, and a lack of institutional support (Scherer and Bertram, 2024; Nührenbörger et al., 2025). The effective implementation of inclusive mathematics education is closely related not only to teachers’ perceptions of diverse learning needs but also to their competencies in teaching mathematics, their attitudes toward inclusive education, and their ability to implement inclusive practices (Mazı, 2026; Charitaki et al., 2026). It demonstrates that teachers’ beliefs about inclusive education can be a significant determinant of inclusive practices and teachers’ assessments of student achievement (Woodcock, 2020). When evaluated from the perspective of Universal Design for Learning (UDL), teachers’ use of multiple representations and flexible teaching methods can be seen as an effort to reduce barriers to participation in mathematics. However, current findings indicate that these practices are generally reactive that is, teachers are limited to responding to situations where students are experiencing difficulties. In other words, rather than designing learning environments that take student diversity into account in advance, teachers are more inclined to modify the instructional process only after students have encountered problems.

Taken together, the findings demonstrate that teachers use a range of instructional approaches consistent with recommendations in the literature. However, the effectiveness of these approaches depends on their systematic implementation, alignment with students’ specific needs, and integration within a comprehensive instructional framework.

### 6.3 Teachers’ views on topics in mathematics associated with learning difficulties

The findings of this study indicate that teachers perceive the most challenging areas for students with mathematics learning difficulties as basic arithmetic operations, problem solving, geometry, and data analysis. These findings suggest that teachers recognize difficulties not only in procedural skills but also in broader mathematical processes requiring reasoning, representation, and conceptual understanding. Teachers’ emphasis on difficulties in basic operations is consistent with previous research on mathematics learning difficulties. Students experiencing mathematical difficulties often demonstrate persistent challenges in number sense, calculation fluency, and understanding numerical relationships (Butterworth et al., 2011; Szűcs and Goswami, 2013). Difficulties in these foundational skills may influence students’ ability to progress toward more complex mathematical concepts because higher-level mathematical learning requires the integration of basic numerical competencies. The participants also highlighted problem solving as one of the most difficult areas for students with mathematics learning difficulties. This finding is consistent with research indicating that these students may experience challenges in understanding problem situations, selecting appropriate strategies, monitoring solution processes, and transferring knowledge to unfamiliar contexts (Fuchs et al., 2012; Stevens et al., 2017). However, problem-solving difficulties should not be interpreted solely as student-related limitations. Instructional approaches, opportunities for mathematical discussion, and teachers’ emphasis on conceptual understanding also influence students’ problem-solving development (Lee et al., 2020). Teachers’ identification of geometry as a challenging area is also meaningful because geometric learning requires spatial reasoning, visualization, and the ability to interpret multiple representations. Previous studies have shown that students with mathematics learning difficulties may experience difficulties in visuospatial processing and representing mathematical relationships (Szűcs and Goswami, 2013). Therefore, the use of visual models, manipulatives, and technology-supported representations may help students develop stronger conceptual understanding in geometry. Another important finding concerns students’ difficulties in data analysis and interpretation. Although data literacy has become an increasingly important component of mathematics education, students with learning difficulties may require additional support in interpreting graphs, organizing information, and making evidence-based conclusions. This suggests that mathematics learning difficulties should not be considered limited to arithmetic skills but should also be examined within broader mathematical competencies.

Overall, the findings indicate that teachers have considerable awareness of the areas in which students experience mathematical difficulties. However, the literature emphasizes that these difficulties emerge from interactions among students’ cognitive characteristics, instructional practices, and learning environments (Nelson et al., 2022). Therefore, supporting students with mathematics learning difficulties requires approaches that address both individual learning needs and the quality of instructional experiences.

### 6.4 Teachers’ views on their professional competencies regarding students experiencing difficulties in mathematics

A significant finding of this study is that teachers do not perceive themselves as sufficiently competent in supporting students with mathematics learning difficulties. Participants expressed a need for professional guidance, collaboration with experts, and additional training opportunities. This finding highlights the importance of teacher competence as a central component of inclusive mathematics education. Previous research has demonstrated that teachers’ knowledge and beliefs regarding mathematics learning difficulties influence their instructional decisions and their ability to provide appropriate support (Hamukwaya and Haser, 2021). Teachers who have limited knowledge about learning difficulties may experience challenges in identifying students’ needs, adapting instructional strategies, and implementing evidence-based interventions. The findings are also consistent with research emphasizing the role of teacher professional development in improving inclusive educational practices. Effective professional development programs can enhance teachers’ confidence, pedagogical knowledge, and ability to implement differentiated instruction for diverse learners (Joswick et al., 2023). However, professional development should not be limited to theoretical knowledge; teachers require opportunities to apply strategies in authentic classroom contexts and receive continuous feedback. The participants’ emphasis on collaboration with experts and colleagues further demonstrates the importance of professional learning communities. Collaborative models enable teachers to share experiences, discuss instructional challenges, and develop context-specific solutions. Research suggests that ongoing professional support and mentoring are more effective than short-term training programs because they provide continuous opportunities for reflection and improvement (Schmidt et al., 2023). Another important implication of the findings is that teacher competence involves more than knowing instructional strategies. Teachers also need awareness of students’ emotional experiences, expectations, and social needs. Students with mathematics learning difficulties may experience reduced confidence and negative attitudes toward mathematics as a result of repeated academic difficulties. Therefore, teachers’ ability to create supportive and inclusive classroom environments is essential for improving both academic engagement and achievement.

Overall, this study advances existing research by shifting the understanding of mathematics learning difficulties from an individual student problem toward a relational and contextual perspective. The findings demonstrate that teachers’ beliefs, professional knowledge, instructional practices, and educational conditions jointly shape how students experiencing mathematics learning difficulties are supported. By highlighting teachers’ experiences within authentic classroom contexts, this study provides important implications for strengthening inclusive mathematics education through improved teacher preparation, professional development, and systemic support.

## 7 Conclusions and implications for practice

This study provides an in-depth examination of mathematics teachers’ beliefs, perceptions, and instructional responses toward students experiencing mathematics learning difficulties (MLD) within the framework of inclusive mathematics education. The findings demonstrate that teachers do not perceive mathematics learning difficulties merely as student-related deficiencies; rather, they understand these difficulties as complex phenomena emerging from the interaction among students’ cognitive characteristics, affective experiences, instructional practices, and contextual conditions. A central contribution of this study is the identification of the complex relationship between teachers’ beliefs and their instructional responses. Although teachers expressed strong commitment to supporting students with mathematics learning difficulties and reported using various adaptive strategies, including individualized instruction, concrete representations, technology-supported activities, and peer-assisted learning, their practices were often constrained by limited professional preparation and insufficient institutional support. These findings suggest that positive attitudes toward inclusion, while necessary, are not sufficient for effective inclusive mathematics teaching. Teachers require systematic opportunities to develop specialized knowledge, practical skills, and confidence in addressing diverse mathematical learning needs. The study also contributes theoretically by extending current understandings of mathematics learning difficulties beyond an individual deficit perspective. By integrating inclusive mathematics education and Universal Design for Learning perspectives, the findings highlight that learning difficulties should be examined within the relationship between learners, teachers, and learning environments. This perspective shifts attention from asking why students fail in mathematics toward examining how educational systems can create more accessible and supportive learning opportunities. From a practical perspective, the findings highlight the need for teacher education programs and professional development initiatives that move beyond general awareness of inclusion. Effective preparation should include classroom-based experiences, strategies for identifying mathematical learning difficulties, differentiated mathematics instruction, and evidence-based intervention approaches. In addition, educational policies should provide schools with adequate resources, collaborative support structures, and opportunities for teachers to work with specialists in developing inclusive practices.

In conclusion, supporting students with mathematics learning difficulties requires a multidimensional approach that recognizes the interconnected roles of students, teachers, instructional practices, and educational contexts. By foregrounding teachers’ professional experiences, this study contributes to a deeper understanding of how inclusive mathematics education can be strengthened and provides important directions for research, teacher education, and educational policy.

The findings of this study have several important implications for mathematics education.

First, teacher education programs should provide more comprehensive preparation regarding mathematics learning difficulties. Pre-service and in-service teachers should be supported in developing knowledge about the cognitive, emotional, and instructional dimensions of mathematics learning difficulties rather than focusing only on students’ academic performance. Second, professional development programs should emphasize evidence-based instructional approaches, including differentiated instruction, explicit teaching strategies, concrete and visual representations, and technology-supported learning environments. Such programs should include practical classroom applications rather than being limited to theoretical explanations. Third, schools should develop collaborative support systems involving mathematics teachers, special education professionals, school counselors, and families. Collaboration among stakeholders may help teachers better understand students’ needs and develop more effective instructional responses. Fourth, technology integration should be approached from a pedagogical perspective. Digital tools should not be considered as independent solutions but should be incorporated into mathematics instruction in ways that promote conceptual understanding, active participation, and individualized learning opportunities. Finally, mathematics classrooms should be designed according to inclusive education principles. Creating supportive learning environments where students with mathematics learning difficulties can participate actively and experience success may contribute to improving both academic achievement and mathematical confidence.

## 8 Recommendations and future research

1. Mathematics teacher education programs should include compulsory courses focusing on identifying and supporting students with mathematics learning difficulties.
2. Professional development programs should provide teachers with practical training on differentiated instruction, evidence-based intervention strategies, and classroom adaptation techniques.
3. Mathematics instruction should incorporate concrete materials, visual representations, and technology-supported activities to facilitate students’ understanding of abstract concepts.
4. Individualized learning opportunities should be developed according to students’ specific needs, while ensuring that these supports are integrated into inclusive classroom practices.
5. Schools should establish collaborative professional support mechanisms that enable mathematics teachers to work with special education experts and other professionals.
6. Future research should examine mathematics learning difficulties through mixed-method and longitudinal designs by including teacher, student, and classroom-level variables.

Future research involving teachers from different regions, school types, and educational levels may provide broader insights into mathematics teachers’ perspectives regarding learning difficulties. Second, the study was based primarily on teachers’ self-reported perceptions and experiences. Although teacher interviews provide valuable insights into classroom practices, future studies may strengthen understanding by incorporating additional data sources, such as classroom observations, student perspectives, instructional materials, and longitudinal achievement data. Third, this study focused on teachers’ perceptions of students who experience difficulties in mathematics rather than formally diagnosed learning disabilities. Future research may examine differences between teachers’ perceptions of mathematics difficulties and students with professionally identified learning disabilities. Future studies may also investigate the effectiveness of professional development programs designed specifically to improve mathematics teachers’ knowledge and self-efficacy regarding learning difficulties. In addition, intervention studies examining the long-term effects of differentiated instruction, technology-supported learning, and collaborative teaching models may provide further evidence regarding effective practices for supporting students with mathematics learning difficulties.

## 9 Limitations

This study has several limitations that should be considered when interpreting the findings. First, the participants consisted of 20 mathematics teachers working in a specific educational context in Turkey’s Eastern Anatolia Region. Therefore, the findings are context-dependent and should not be interpreted as statistically generalizable to all mathematics teachers.

## Data Availability

The raw data supporting the conclusions of this article will be made available by the authors, without undue reservation.
